# Balance Screening of Vestibular Function in Subjects Aged 4 Years and Older: A Living Laboratory Experience

**DOI:** 10.3389/fneur.2017.00631

**Published:** 2017-11-28

**Authors:** María Carolina Bermúdez Rey, Torin K. Clark, Daniel M. Merfeld

**Affiliations:** ^1^Jenks Vestibular Physiology Laboratory, Massachusetts Eye and Ear Infirmary, Boston, MA, United States; ^2^Otolaryngology, Harvard Medical School, Harvard University, Boston, MA, United States; ^3^Smead Aerospace Engineering Sciences, University of Colorado, Boulder, CO, United States; ^4^Otolaryngology, The Ohio State University, Columbus, OH, United States

**Keywords:** balance, aging, vestibular, screening, falls

## Abstract

To better understand the various individual factors that contribute to balance and the relation to fall risk, we performed the modified Romberg Test of Standing Balance on Firm and Compliant Support, with 1,174 participants between 4 and 83 years of age. This research was conducted in the Living Laboratory^®^ at the Museum of Science, Boston. We specifically focus on balance test condition 4, in which individuals stand on memory foam with eyes closed, and must rely on their vestibular system; therefore, performance in this balance test condition provides a proxy for vestibular function. We looked for balance variations associated with sex, race/ethnicity, health factors, and age. We found that balance test performance was stable between 10 and 39 years of age, with a slight increase in the failure rate for participants 4–9 years of age, suggesting a period of balance development in younger children. For participants 40 years and older, the balance test failure rate increased progressively with age. Diabetes and obesity are the two main health factors we found associated with poor balance, with test condition 4 failure rates of 57 and 19%, respectively. An increase in the odds of having fallen in the last year was associated with a decrease in the time to failure; once individuals dropped below a time to failure of 10 s, there was a significant 5.5-fold increase in the odds of having fallen in the last 12 months. These data alert us to screen for poor vestibular function in individuals 40 years and older or suffering from diabetes, in order to undertake the necessary diagnostic and rehabilitation measures, with a focus on reducing the morbidity and mortality of falls.

## Introduction

Falls are a leading cause of injury and death throughout the world. In 2003 in the US, fatal falls or hospitalizations for hip fractures occurred at a rate of approximately 36.8 per 100,000 people aged 65 and older (1). According to the World Health Organization (WHO) (2), “*falls and consequent injuries are major public health problems*. … *Falls lead to 20–30% of mild to severe injuries, and are the underlying cause of 10–15% of all emergency department visits*. … *Falls [also] account for 40% of all injury deaths*.”

Mobility changes with age are complex [e.g., (3)]. The WHO notes that biological factors, including age, interact with behavioral, environmental, and socioeconomic risks to yield fall risk (2). As a pertinent example for the data reported herein, postural control (i.e., “balance”) results from a combination of various subcomponents, such as biomechanics, movement strategies, sensory function, sensory integration, cognitive processing, etc. (4)—each of which can individually (or in combination) be impacted by dysfunction. Therefore, interventions ideally will vary by the individual case. For example, fall prevention for someone who suffers from poor vestibular function (e.g., vestibular hypofunction) will likely differ—at least in part—from fall prevention for someone with poor proprioception.

We focus herein on vestibular contributions to balance and falls because, as noted earlier: (a) the vestibular system is fundamental for balance (5–11) and (b) recent data show large increases in vestibular perceptual thresholds begin after age 40 (12). Data from patients with vestibular disorders demonstrate the crucial role that the vestibular system plays for balance control (5–11) as do some studies that combine vestibular modeling with empirical balance data (8, 13, 14).

Because balance testing provides a screening test for all vestibular organs (e.g., semicircular canals and otoliths) as well as central sensory integration, one standard screening used to approximate the prevalence of vestibular dysfunction is the fourth condition of a modified Romberg Test of Standing Balance on Firm and Compliant Support Surfaces (modified Romberg Test) (15, 16). Subjects must rely on their vestibular system for test condition 4 (C4), because the contribution of visual and kinesthetic cues are eliminated/reduced by closing the eyes and standing on thick memory foam, respectively. Therefore, failure to complete C4 of this modified Romberg test provides a proxy for vestibular dysfunction.

This modified Romberg test was performed during the National Health and Nutrition Examination Survey (NHANES) between 2001 and 2004, on US adults aged 40 years and older. Data show a C4 failure rate of 35.4% (15). In contrast, in a similar population study of 3,267 Korean adults aged 40 or older, data show a C4 failure rate of just 1.84% (16). The main methodological difference between these two balance studies was that for the American study, balance testing was performed with feet together (15), while for the Korean study, participants were asked to stand with their feet 10 cm apart (16), which provided a broader base of stability.

The US study reported that subjects who failed the same C4 test condition were 6.3 times more likely to have fallen in the past year (15). While speculative, our recent article showed calculations that combined the findings of these (and other) studies to suggest that 48,000 to 152,000 accidental deaths in the US each year might correlate with vestibular dysfunction (12). Even the lowest estimate, which would place this as the 10th leading cause of death, conveys the importance of understanding vestibular contributions to falls in otherwise healthy, but aging humans.

We are aware of only one earlier population study that performed balance testing focused on vestibular contributions for subjects younger than 20 years (17). This study included subjects between the age of 7 and 81. For sensory organization test number 6 (SOT6), which is considered a vestibular test condition, data showed a broad balance performance plateau between the ages of about 20 and 50 with performance degrading below the age of 20 and above the age of 50. However, this study did not correlate balance and fall history.

Given that there are no recent studies analyzing the vestibular contributions to balance in both children and adults, we intend to build upon the Agrawal study by testing a broader age group (participants 4 years of age and older) using the same modified Romberg test (15). We decided to use the version of the modified Romberg Test in which testing was performed with feet together because this is the one that has been used more extensively in the US population (15, 18, 19), it is more challenging than when performed using a wider stance (16), and it is the one that our group previously used to correlate with vestibular perceptual threshold data (12).

## Materials and Methods

This research was conducted in the Living Laboratory^®^ at the Museum of Science, Boston, where all participants were museum visitors. Living Laboratory^®^ is an innovative model for educating the public about human health and behavior. Museum visitors have the chance to engage in one-on-one conversations with scientists from their own community and participate in active research studies (20). During our time at the museum, one of the goals was to increase awareness of balance, including the importance of a normal and functional vestibular system for daily living (e.g., for balance).

All interested museum visitors aged 4 years or older were eligible to participate. For minors, the presence of a legal guardian was required. Subjects were excluded from balance testing if they were unable to stand unassisted, weighed more than 275 pounds, had a foot or leg amputation, were dizzy or lightheaded at the time of the test, or had a waist circumference that could not accommodate our largest standard safety gait belt (approximately 150 cm). Details on the number of subjects excluded are provided in the Section “Results.”

Informed consent was obtained from all subjects or their guardians as dictated by the Declaration of Helsinki. A waiver of documentation of consent was approved by the institutional ethical committee at the Massachusetts Eye and Ear Infirmary and the Museum of Science, since providing name and signature on the informed consent form would have provided the only Personally Identifiable Information collected for this study.

If eligible, participants filled out a questionnaire collecting demographic information (age, race, ethnicity, education), two questions regarding history of falls and dizziness in the last year, and cardiovascular risks. Subsequently subjects performed the modified Romberg test (details below). All study data were collected and managed using REDCap™ (Research Electronic Data Capture) electronic data capture tools hosted at the Massachusetts Eye and Ear Infirmary. REDCap™ is a secure, web-based application designed to support data capture for research studies (21). The Supplementary Material includes the exact text that the subjects responded to *via* a REDCap™ link. To avoid any impact of balance testing on subjective reports, the questionnaire was always completed prior to the balance test. Details of each of these aspects are provided below.

### Questionnaire

Exact wording of the questionnaire is provided in the Supplementary Material; here we provide a summary of the questions/items. Sex was grouped as male or female. Age at interview was collected in years. Height was recorded in feet and inches and weight in pounds. Ethnicity was grouped as American Indian/Alaska Native, Asian, White, Native Hawaiian or other Pacific Islander, Black or African American, and more than one race, following the NIH suggestion. In addition, participants were asked if they identified themselves as Hispanic/Latino or not. To estimate the presence of hypertension and diabetes, the following questions were used (each with yes/no answers): Do you have hypertension/high blood pressure? Do you take any blood pressure medication? Do you have diabetes/high blood sugar? Do you take any medication to lower blood sugar?

For subjects aged 18 or older, educational information, current smoking, pregnancy, and history of dizziness or balance impairment were also collected. Education was grouped as some high school, high school graduate including General Equivalency Diploma, some college, college graduate, and advanced degree. Current smoking included the number of years smoked and the current number of cigarettes smoked per day. In case a woman indicated she was pregnant, weeks of pregnancy were registered. Balance and dizziness was assessed *via* two questions: “during the past 12 months, have you had dizziness or difficulty with balance?” and “during the past 12 months, have you had difficulty with falling?”

### Balance Testing

The modified Romberg test we used had four conditions. Each condition must be passed in order to move to the next condition. All conditions were performed standing with feet together and arms crossed. To pass the first condition (C1), each participant had to stand on the floor (firm surface) for 15 s with eyes open. To pass the second condition (C2), they had to stand on the floor for 15 s with eyes closed. To pass the third condition (C3), they had to stand on memory foam with eyes open for 30 s. To pass the final condition (C4), they had to stand on the foam with eyes closed for 30 s. This C4 test condition primarily assesses vestibular function, since visual contributions are eliminated and the foam makes kinesthetic cues unreliable (15, 22). The balance test was scored on a pass/fail basis. Failure was defined as participants needing to open their eyes, move their feet or arms to maintain stability, or be supported by the experimenter to prevent a fall before the timed trial duration. All subjects were allowed up to two attempts to complete each condition. For the two conditions (C3 and C4) requiring a foam pad, a Sunmate medium density foam pad (16 in × 18 in × 3 in") was used. Testing was performed by one of the authors (María Carolina Bermúdez Rey or Torin K. Clark).

### Statistical Analysis

We calculated the prevalence of failing the C4 balance test condition, which serves as a proxy for vestibular dysfunction. Therefore, we will sometimes refer to the C4 balance test condition as the “vestibular test condition.” A Fisher’s exact test was used to test for prevalence differences. Multiple logistic regression was used to estimate the odds of failing the vestibular condition in association with various sociodemographic and cardiovascular risk factors and to estimate the odds of reporting a fall associated with failing the vestibular condition. We fit our multiple logistic regression model mimicking the approach of Agrawal et al. (15), except where inappropriate (e.g., educational level was not included in the model fits with child and adolescent subjects). Collinearity between predictor variables was assessed with Chi-squared tests of association. Adjusting for multiple comparisons, significant associations were not found for most predictor variables; exceptions are noted in the Discussion. Five percent (*p* < 0.05) was the statistical criteria applied throughout. Analyses were performed using SAS statistical software (SAS Institute Inc., Cary, NC, USA).

## Results

**Figure 1:**
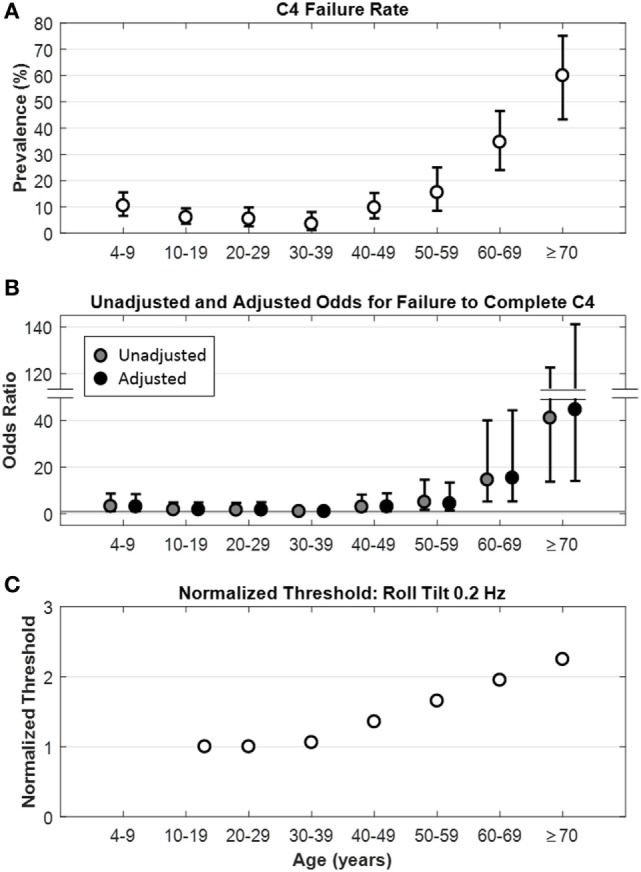
Relationship between age and C4 failure. **(A)** Rate of C4 failure as a function of age (*p* < 0.0001), whiskers show ±95% confidence intervals. **(B)** Unadjusted (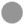) and adjusted (●) odds ratios for failure to complete C4 by age. Whiskers show ±95% confidence intervals. Gray horizontal line is the line of null effect. Odds ratios were adjusted for sex, age, race, ethnicity, and history of diabetes and hypertension. **(C)** Model fit for roll tilt 0.2 Hz thresholds as a function of age in healthy participants, from Bermúdez Rey et al. (12).

A total of 1,227 museum goers filled out the initial questionnaire. Of these, 27 were excluded and 26 withdrew before completing the study, leaving a sample of 1,174 participants. Of these 1,174, none failed to complete conditions C1, C2, or C3. The overall prevalence of failure to complete C4 in this Living Laboratory experience was 11.24% (Table 1). The C4 failure rate was stable between 10 and 39 years of age, with a slight increase in the failure rate for participants 4–9 years of age. Above 40 years of age, the C4 failure rate increased significantly with age (Figure 1A). Among all adults (aged 18 years and older), the C4 failure rate was 12.65% [95% confidence interval (CI): 10.36–15.24%], while it was 21.70% (95% CI: 17.58–26.30%) for participants aged 40 years and older.

**Table 1:** C4 failure rate by demographic factors and unadjusted and adjusted odds of failing C4 for participants 4 and older.

Characteristic	No. (%) of participants	Prevalence of C4 failure (95% CI) (%)	*p*-Value	C4 failure	
				Unadjusted OR (95% CI)	Adjusted OR (95% CI)^a^
All participants	1,174	11.24 (9.49–13.19)	–		
**Sex**					
Male	465 (39.61)	13.33 (10.38–16.76)	0.0728	1 (reference)	1 (reference)
Female	709 (60.39)	9.87 (7.78–12.31)		0.71 (0.49–1.04)	**0.59 (0.39–0.89)**
**Age (years)**					
4–9	201 (17.12)	10.45 (6.58–15.53)	**<0.0001**	**3.20 (1.18–8.69)**	**3.04 (1.09–8.44)**
10–19	283 (24.11)	6.01 (3.54–9.44)		1.75 (0.63–4.84)	1.73 (0.61–4.90)
20–29	184 (15.67)	5.43 (2.64–9.77)		1.57 (0.53–4.71)	1.65 (0.54–4.98)
30–39	142 (12.10)	3.52 (1.15–8.03)		1 (reference)	1 (reference)
40–49	165 (14.05)	9.70 (5.64–15.27)		**2.94 (1.05–8.24)**	**3.04 (1.05–8.74)**
50–59	84 (7.16)	15.48 (8.51–25.01)		**5.01 (1.72–14.62)**	**4.37 (1.43–13.40)**
60–69	75 (6.39)	34.67 (24.04–46.54)		**14.53 (5.29–39.99)**	**15.35 (5.32–44.33)**
≥70	40 (3.41)	60.00 (43.33–75.14)		**41.01 (13.76–122.62)**	**44.60 (14.09–141.17)**
**Race**^b^					
White	987 (85.01)	11.35 (9.44–13.49)	0.5795	1 (reference)	1 (reference)
Black or African American	25 (2.15)	8.00 (0.98–26.03)		0.68 (0.16–2.92)	0.51 (0.10–2.76)
Asian	80 (6.89)	7.50 (2.80–15.61)		0.63 (0.27–1.49)	0.75 (0.28–2.00)
Other (American Indian, Hawaiian, and more than one race)	69 (5.94)	14.49 (7.17–25.04)		1.32 (0.66–2.66)	1.33 (0.60–2.94)
**Ethnicity**^c^					
Non-Hispanic	1,097 (94.24)	10.94 (9.15–12.94)	0.1651	1 (reference)	1 (reference)
Hispanic	67 (5.76)	16.42 (8.49–27.48)		1.61 (0.74–3.22)	2.18 (0.99–4.90)

*^a^Adjusted for sex, age, race, ethnicity, and history of diabetes, and hypertension*.

*^b^Data were missing for 13 participants*.

*^c^Data were missing for 10 participants*.

*Bold text identifies a statistically significant finding. The *p* value shown for age (*p* < 0.0001) is the result of a statistical test comparing all age groups in the same test. When analyzed separately (as pairs), there was no significant difference in the prevalence of C4 failure for the following pairs: 4–9 vs. 10–19, 4–9 vs. 20–29, 4–9 vs. 40–49, 4–9 vs. 50–59, 10–19 vs. 20–29, 10–19 vs. 30–39, 10–19 vs. 40–49, 20–29 vs. 30–39, 20–29 vs. 40–49, and 40–49 vs. 50–59 years (*p* values between 0.09 and −0.84). The other 16 pairs show a significant difference in the prevalence of C4 failure, with *p* values ranging from 0.04 to 0.00000001*.

*OR, odds ratio*.

When looking at the influence of demographic characteristics, C4 failure rates did not differ by sex, race, or ethnicity (Table 1), nor did they differ by education level for adults aged 40 years and older (Table 2). Regarding cardiovascular risk factors in adults, C4 failure rate was significantly increased in individuals with a history of hypertension or a history of diabetes for the overall population, and a significant difference by body mass index (Table 3). There was no difference between smokers and non-smokers. Adults who reported a history of dizziness were more likely to fail C4, as were adults who reported falling in the past year (Table 3).

**Table 2:** C4 balance test failure rate by educational level in adults 40 and older.

Characteristic	Number of participants	C4 balance test, % failure (95% CI)	*p*-Value
All participants	364	21.70 (17.58–26.30)	–
**Educational level**			
Some high school	6 (1.65)	33.33 (4.33–77.72)	0.547
High school graduate including GED	15 (4.13)	33.33 (11.82–61.62)	
Some college	78 (21.49)	24.36 (15.35–35.40)	
College graduate	116 (31.96)	18.97 (12.28–27.29)	
Advanced degree	148 (40.77)	20.95 (14.70–28.39)	

**Table 3:** C4 failure rate and odds for cardiovascular factors for participants 18 and older.

Characteristic	No. (%) of participants	Prevalence of C4 failure (95% CI) (%)	*p-*Value	C4 failure	
				Unadjusted OR (95% CI)	Adjusted OR (95% CI)^a^
All participants	751	12.65 (10.36–15.24)	–		
**History of hypertension**^b^					
No	667 (88.93)	10.49 (8.27–13.07)	< **0.0001**	1 (reference)	1 (reference)
Yes	83 (11.07)	30.12 (20.53–41.18)		**3.68 (2.06–6.40)**	0.83 (0.41–1.69)
**History of diabetes mellitus**^b^					
No	736 (98.13)	11.82 (9.58–14.38)	**<0.0001**	1 (reference)	1 (reference)
Yes	14 (1.87)	57.14 (28.86–82.34)		**9.95 (2.93–35.46)**	**4.29 (1.15–15.99)**
**Smokers**^c^					
No	713 (95.19)	12.62 (10.27–15.29)	0.7973	1 (reference)	1 (reference)
Yes	36 (4.81)	13.89 (4.67–29.50)		1.12 (0.33–3.00)	2.13 (0.70–6.44)
**BMI (kg/m^2^)**					
<25	402 (53.53)	9.45 (6.78–12.74)	**0.0116**	1 (reference)	1 (reference)
25–30	253 (33.69)	15.42 (11.20–20.46)		**1.75 (1.08–2.81)**	1.36 (0.78–2.39)
30≤	96 (12.78)	18.75 (11.51–28.00)		**2.21 (1.20–4.08)**	**2.35 (1.11–4.98)**
**Self–reported dizziness**^b^					
No	666 (88.80)	11.56 (9.23–14.24)	**0.0146**	1 (reference)	1 (reference)
Yes	84 (11.20)	21.43 (13.22–31.74)		**2.09 (1.10–3.78)**	1.36 (0.66–2.77)
**History of falls**					
No	734 (97.87)	12.26 (9.98–14.86)	**0.041**	1 (reference)	1 (reference)
Yes	16 (2.13)	31.25 (11.02–58.66)		**3.25 (0.86–10.42)**	2.21 (0.53–9.17)

*^a^Adjusted for sex, age, race, ethnicity, BMI, smoking status, as well as diabetes and hypertension history*.

*^b^Data were missing for one participant*.

*^c^Data were missing for two participants*.

*Bold text identifies a statistically significant finding*.

*OR, odds ratio*.

To take into account the potential effect of demographic characteristics and cardiovascular risk factors on the association between age and C4 failure, multivariate analyses were performed, noting a persistent influence of age on the odds of not completing C4 (“Adjusted OR” in Table 1 and Figure 1B). Analyses were performed in the overall population as well as in two subsamples: participants aged 18 years and older and those 40 years and older. For all three groups, increasing age was associated with increased odds of not completing C4; due to the similarity of the results, we only show data for the overall population (Table 1).

**Figure 2:**
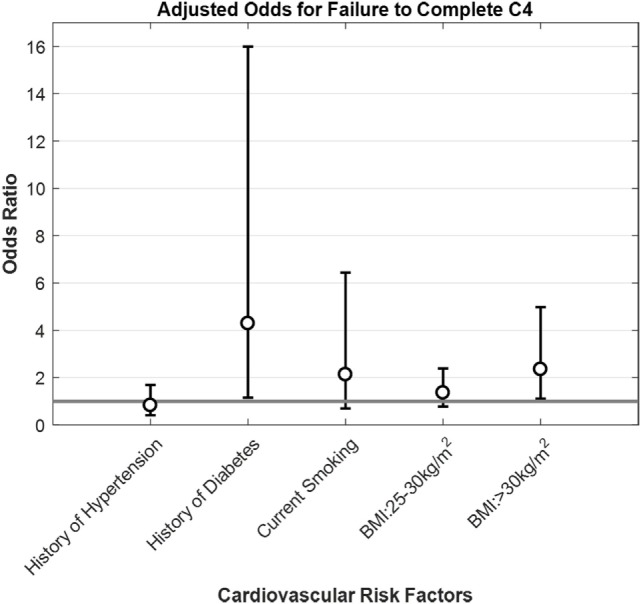
Adjusted odds for failure to complete C4 by cardiovascular factors for participants 18 and older. Opened circles (∘) show odds ratio for each condition, whiskers show ±95% confidence intervals and gray horizontal line is the line of null effect. Odds ratios were adjusted for sex, age, race, ethnicity, BMI, smoking status, diabetes, and hypertension history.

A self-reported history of diabetes was associated with a significant increase in the odds of not completing C4 in adjusted analyses (Figure 2), both for the overall population and the two subsamples (Table 3 shows the subsample of adults). The C4 failure rate among adults with diabetes was 57% (95% CI: 29–82%).

Surprisingly, females had significantly lower odds of not completing C4 (i.e., increased odds of completing C4) in the multivariate analyses adjusting for age, race ethnicity and cardiovascular risk factors (Table 1). In our sample, adult females were significantly associated with having lower body mass index, which we further consider in the section “Discussion.”

History of hypertension, smoking, self-reported dizziness and history of falls did not increase the odds of not completing C4 following adjusted analyses, both for the overall population and the two subsamples (Table 3 shows the subsample of adults). Having a history of hypertension was significantly associated with age, self-reported dizziness, and body mass index. When we refit the model without a history of hypertension as a predictor, it did not impact the significance of the other variables (not shown). For adults, failure to complete C4 was similarly not associated with an increase in the odds of self-reported dizziness or history of falls, even after adjusted analyses (Table 4).

**Table 4:** Prevalence and odds of self-reported dizziness and history of falls for participants who passed and failed the vestibular balance test condition (C4).

	Self-reported dizziness			History of falls		
C4 balance test failure	Prevalence	Unadjusted OR	Adjusted OR^a^	Prevalence	Unadjusted OR	Adjusted OR^a^
No	10.08 (7.88–12.64)	1 (reference)	1 (reference)	1.68 (0.84–2.98)	1 (reference)	1 (reference)
Yes	18.95 (11.63–28.28)	**2.09 (1.10**–**3.78**)	1.39 (0.69–2.80)	5.26 (1.73–11.86)	3.25 (0.86–10.42)	2.0 (0.47–8.50)

*^a^Adjusted for sex, age, race, ethnicity, BMI, smoking status, and history of diabetes and hypertension*.

*Bold text identifies a statistically significant finding*.

*OR, odds ratio*.

**Figure 3:**
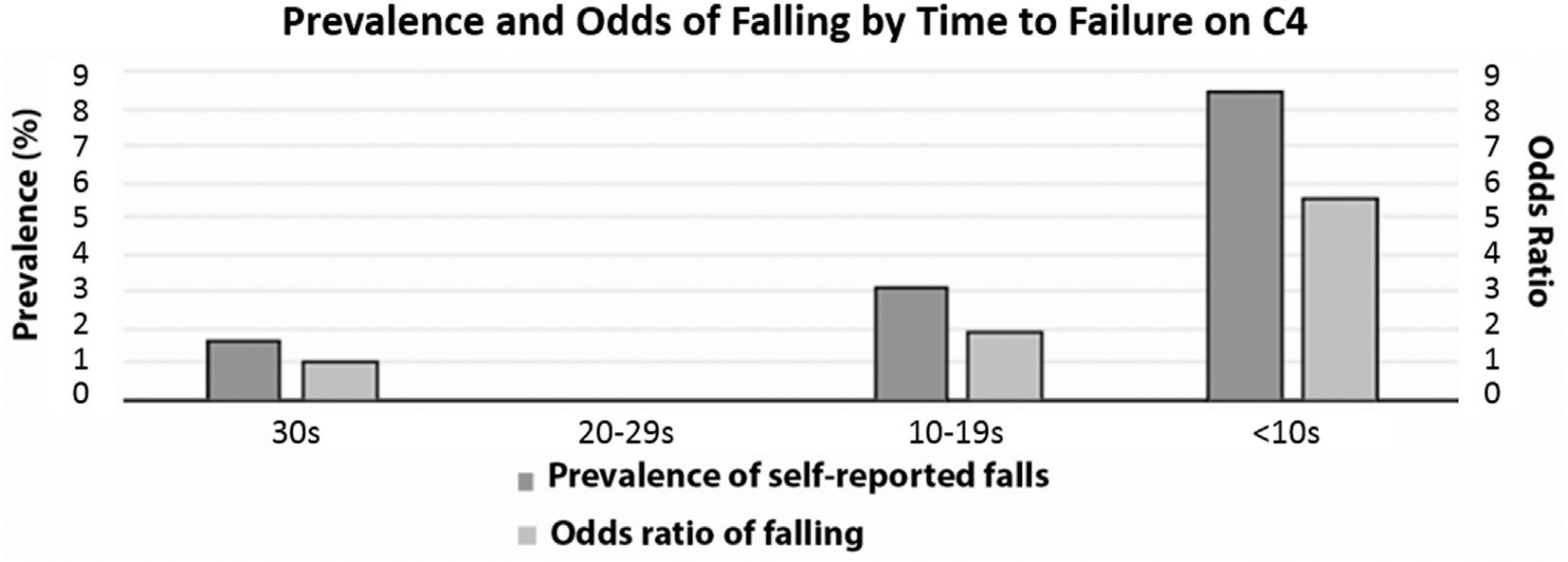
Prevalence and odds of falling by time to failure of C4. Dark gray bars show % of C4 failure and light gray bars show odds ratio of falling. None of the 16 participants who failed C4 between 20 and 29 s self-reported falls in the last 12 months.

When looking at time to failure in the last test condition (where 30 s corresponds to passing C4), there was no statistically significant difference between age groups (*p* = 0.9121, data not shown). However, for adults, as the time to failure shortened, there was a significant increase in both the prevalence and the odds of falling (defined as self-reported history of falling in the last year) (Figure 3). Only 1.7% of adults who passed C4 by maintaining balance for 30 s reported falls in the previous year, whereas for those who were only able to maintain balance for less than 10 s, fall prevalence was 8.5%—five times higher than for participants who passed C4 (Table 5). Consistent with this prevalence finding, the odds of falling increased as the time to failure shortened, reaching statistical significance only for participants who failed in less than 10 s, having a 5.6-fold increase in the odds of falling (Table 5).

**Table 5:** Prevalence and odds of falling with time to failure on C4.

Time to failure (s)	N	Prevalence (95% CI)	OR (95% CI)
30	656	1.7 (0.8–3.0)	1 (reference)
20–29	16	0	ND
10–19	32	3.1 (0.1–16.2)	1.9 (0.2–15.1)
<10	47	8.5 (2.4–20.4)	**5.5 (1.7–17.9)**

*Bold text identifies a group that is statistically significantly different from the reference group*.

*OR, odds ratio; ND, no data*.

## Discussion

These findings suggest that vestibular contributions to balance are relatively stable between the ages of 10–39, with the lowest C4 failure rate for participants 30–39 years of age (3.52%). As a novel finding, there was a slightly higher C4 failure rate (10.45%) for participants 4–9 years of age, presumably representing the development of balance in younger individuals. Above age 40, as previous studies have shown (15, 16, 19), the C4 failure rate increased markedly and progressively, reaching a rate of 60% above age 70, making balance deficits due to poor vestibular function fairly common. This general pattern also occurred in an earlier study of balance using the computerized posturography SOT6 as the “vestibular condition” (17). Furthermore, our rate of C4 failure for adults aged 40 years and older (21.70%) is comparable with the 35% rate reported from the NHANES study (15), as is the pattern of increase seen as participants become older. Our lower C4 failure rate may have resulted from selection bias, owing to the general degree of health required for someone to be able to visit a museum in comparison to the health of the general population.

Here, we find the vestibular contributions to balance degrade with age above 40. We have previously shown that vestibular perceptual thresholds increase with age above the age of about 40 (Figure 1C) (12). These two data sets support the idea that only after age 40 do age effects on the vestibular system become substantial enough to alter balance function or vestibular perception thresholds in the general adult population.

Our recent study also showed that variations in vestibular function measured directly in healthy “normals”—specifically, variations in vestibular perceptual roll tilt thresholds—were highly correlated with balance function. Specifically, we reported strong correlations between healthy subjects’ inability to complete C4 with increasing yaw rotation (1.04 vs. 1.43°/s), y-translation (0.69 vs. 1.05 cm/s), z-translation (1.62 vs. 3.67 cm/s), and roll tilt (0.40s vs. 0.76°/s at 0.2 Hz) thresholds (12). These self-motion perceptual thresholds have previously been shown to be 2–50× higher in fully compensated patients suffering total vestibular loss (23), suggesting a dominant role of the vestibular system. Consistent with earlier findings linking age and balance deficits [e.g., (17, 24–26)], we too reported that increasing age was linked to these balance test failures. But even when age was considered as an independent factor, balance test failures were still correlated with higher roll tilt thresholds at both 0.2 and 1 Hz. The fact that only roll tilt thresholds (and not the other thresholds measured) correlated with balance test failures after adjusting for age shows that this is not due to a general vestibular degradation. Rather, the fact that only roll tilt thresholds correlated with C4 failure suggests (but does not prove) a causal contribution since roll tilt vestibular cues are directly relevant to postural control in the roll plane. Furthermore, that the effect was particularly prominent at 0.2 Hz suggests the importance of both the semicircular canals and otoliths, since roll tilt thresholds near this frequency have been shown to require the integration of sensory cues from the canals and otoliths (27).

As we mentioned, females had significantly higher odds of completing C4 in the multivariate analyses adjusting for age, race ethnicity and cardiovascular risk factors. We do not know how to interpret that sex showed a significant effect on the odds of failing C4. Since we chose to use the standard statistical criterion (*p* = 0.05), it is certainly possible that this could be one of the 1 in 20 random effects that happen to appear significant when tested. Alternatively, the museum population could have led to some sort of selection bias—either in who attended the museum or who agreed to participate. For example, we note adult females in our population had a significantly lower body mass index. This association makes it difficult to attribute the reduced odds of failing C4 to either sex or body mass index. Of course, there could be a real effect of sex on balance. Since C4 is the vestibular condition, this would potentially suggest that age-matched women had better vestibular function, but this hypothesis is not consistent with our recent comprehensive testing of vestibular thresholds that found no significant difference in five different vestibular thresholds (12). We are not aware of any other studies establishing substantive sex differences for either balance tasks or vestibular measures.

When we look at health factors associated with a higher rate of C4 failure, self-reported history of diabetes stands out at 57% (95% CI: 29–82%)—a percentage almost as high as the one for individuals 70 years of age and above—and with a 4.3 (95% CI: 1.15–15.99) increase in the odds of failing the C4 test condition, which supports the suggestion that diabetes mellitus is a risk factor for vestibular dysfunction (18, 28, 29). It is well known that diabetics have an increased risk of falling (18, 30), which has been attributed primarily to peripheral neuropathy (31–34). Studies have shown the association between diabetes and altered vestibular tests, like cervical vestibular-evoked myogenic potentials and ocular VEMP (35, 36), head thrust dynamic visual acuity testing (36), vestibulo-ocular reflex, and optokinetic reflex (37).

Furthermore, studies have demonstrated the role of vestibular function in the risk of falling for people suffering diabetes (18, 38). Having determined that diabetes increases the risk of falls, it is even more important to develop a screening tool that allows us to establish if the risk is higher due to poor vestibular function. In the case that this is proven to be true, potentially helpful therapies focused on vestibular rehabilitation (39) could be initiated in a prompt manner, when pertinent. The urgency of this matter is highlighted because of the increased risk of hip, foot, and spine fractures in adults suffering diabetes, that is not attenuated after adjustment for diabetes-related complications (40), thus increasing morbidity and mortality of a potential fall.

A second health factor that deserves a special mention is BMI. In this study, as BMI increases, the rate of failure of C4 increases too (Table 3), going from 9.45% (95% CI: 6.78–12.74%) for people with BMI < 25 kg/m^2^ (normal range) to 18.75% (95% CI: 11.51–28.00%) for individuals with BMI ≥ 30 kg/m^2^ (obese). More importantly, there was a significant 2.4-fold increase in the odds of failing C4 for BMI ≥ 30 kg/m^2^, after adjusting for sex, age, race, ethnicity, smoking status, as well as diabetes and hypertension history. This suggests an association between obesity and poor vestibular function. A large national German study (41), found a significant 1.8-fold increase in the odds of having vestibular vertigo both for individuals categorized as overweight (BMI 25–30 kg/m^2^) or obese (BMI ≥ 30 kg/m^2^). Furthermore, posturography differences between obese subjects and their lean counterparts have been reported by multiple studies, both in children (42, 43) and adults (42, 44–46). Given that more than one-third (36.5%) of US adults and about 17% of US children and adolescents meet obesity criteria (47), an effort to elucidate if obesity might contribute to vestibular dysfunction (e.g., *via* glycation or another mechanism) and to establish if treating obesity improves either vestibular function or balance performance might impact public health.

As in the Agrawal study (15), these data show that individuals who fail C4 were more likely to report having dizziness and a history of falls. In both studies, when performing unadjusted analyses, those who reported a history of falling were significantly more likely to fail C4. Unexpectedly, for our data when we performed the adjusted analysis accounting for other factors, there was no significant increase in the odds of falling for people who fail C4 (Table 3). This differs from the findings reported earlier (15) but may be due to the small number of individuals who reported falls in our study (16 of 751 of participants 18 and older) and/or may be related to a possible sampling bias described earlier whereby individuals who go to the museum may be healthier than a random population sampling. Specifically, while speculative, individuals who have recently (e.g., in the past year) fallen may decide to go to the Museum less frequently than individuals who have not recently fallen. We note that while not significant, the estimated odds for failing C4 was 2.2 for individuals who reported falling in the last year.

We did notice an increase in the odds of having fallen in the last year as performance times on C4 decrease. Specifically, once individuals dropped below a time failure of 10 s, there was a significant 5.5-fold increase in the odds of having fallen (Table 5). This failure time of 10 s is lower than the trial duration identified by a previous study (20 s) as the threshold at which the risk of falling rises (19). Of all people who fail C4, individuals who fall before 10 s might be the ones who benefit the most from therapies that focus on balance enhancement and fall risk reduction, especially if they have diabetes, osteoporosis or other factors that predispose individuals to fractures.

We note that failing C4 of our modified Romberg test only *suggests* poor vestibular function, but is not able to *confirm* the presence of vestibular dysfunction or to diagnose the exact vestibular modality (modalities) affected (i.e., rotational transduced *via* semicircular canals, translational transduced *via* saccule or utricle, or tilt *via* central integration of canal and otolith cues). Thus further correlations between balance abnormalities and objective measures of each vestibular modality, as in our earlier study (12), are imperative. This knowledge can be used for the purpose of better understanding the role of vestibular function for balance and how poor vestibular function contributes to the risk of falls, and to design and test vestibular rehabilitation techniques. Since such clinical vestibular tests take time and require health care resources, which are limited, it is not difficult to envision using a simple balance test as a screen to determine who needs full vestibular function tests (e.g., a comprehensive set of perceptual threshold assays).

## Ethics Statement

This study was carried out in accordance with the recommendations of Massachusetts Eye and Ear Infirmary institutional review board, with informed consent from all subjects. Informed consent was obtained from all subjects or their guardians in accordance with the Declaration of Helsinki; due to the minimal risk characteristics of this study, a waiver of documentation of consent was granted. The protocol was approved by the institutional ethical committee.

## Author Contributions

MB and DM designed the study and assisted in acquisition of data, statistical analyses and interpretation, and manuscript preparation. TC also assisted in acquisition and interpretation of data and manuscript preparation. All authors approved the final version of this article.

## Conflict of Interest Statement

The authors declare that the research was conducted in the absence of any commercial or financial relationships that could be construed as a potential conflict of interest.
