# Supplementary material for: Balance Screening of Vestibular Function in Subjects Aged 4 Years and Older: A Living Laboratory Experience
Source: Front Neurol. 2017 Nov 28;8:631. doi: 10.3389/fneur.2017.00631 (PMC5712334; doi:10.3389/fneur.2017.00631)
Supplement: Supplementary file 1 [file Data_Sheet_1.docx]

# Appendix - Balance Questionnaire

Note: questionnaires were filled out by participants and/or their guardians using an iPad and a REDCap^TM^ interface. For individuals under 18 years of age, questions regarding education level, smoking, pregnancy, history of dizziness or balance impairment were not included.

**BALANCE QUESTIONNAIRE**

Please complete the survey below.

Thank you!

*All subjects*

Gender: ∘ Male ∘ Female

Age __________________________________________________________

(For subjects 89 YEARS AND OLDER, use YEAR OF BIRTH)

Hispanic or Latino? ∘ Yes ∘ No

Ethnicity

∘ American Indian/Alaska Native

∘ Asian

∘ White

∘ Native Hawaiian or Other Pacific Islander

∘ Black or African American

∘ More than one race

SAFETY QUESTIONS

Do you have any amputations of your legs and feet other than toes? ∘ Yes ∘ No

How much do you weigh without shoes? ___________________

(in Lbs)

Can you stand on your own? ∘ Yes ∘ No

Do you have a leg brace? ∘ Yes ∘ No

Are you feeling any dizziness or lightheadedness now? ∘ Yes ∘No

*All subjects who met inclusion criteria*

Education level

∘ < High school

∘ High school diploma, including GED

∘ >High school

(GED = General Equivalency Diploma)

HEIGHT

Please, enter your height in the following fields. Put feet in the first one, and inches in the second one. For example, in you are 5'8'', put 5 in the first field and 8 in the second one.

ft __________________________________

in __________________________________

Do you have hypertension / high blood pressure? ∘ Yes ∘ No

Do you take any blood pressure medication? ∘ Yes ∘ No

Do you have diabetes / high blood sugar? ∘ Yes ∘No

Do you take any medication to lower blood sugar? ∘ Yes ∘ No

Do you smoke? ∘ Yes ∘ No

Number of years smoked __________________________________

Number of cigarettes a day __________________________________

Are you pregnant? ∘ Yes ∘ No

BALANCE QUESTIONS

During the past 12 months, have you had dizziness or difficulty with balance?

∘Yes ∘ No

During the past 12 months, have you had difficulty with falling? ∘ Yes ∘ No
